# Metabolomic and exome sequence analysis reveal novel molecular signatures associated with colorectal cancer relapse

**DOI:** 10.1186/1753-6561-6-S6-P41

**Published:** 2012-10-01

**Authors:** Robinder Gauba, Thanemozhi G Natarajan, Lei Song, Krithika Bhuvaneshwar, Subha Madhavan, Yuriy Gusev

**Affiliations:** 1 Innovation Center for Biomedical Informatics, Georgetown University Medical Center, Washington DC, 20007, USA

## Background

Colorectal cancer (CRC) is the third leading cancer killer in the United States, affecting both men and women. Standard treatment for stage II colon cancer is surgical removal of the cancer and an area surrounding the cancer. Adjuvant chemotherapy may also be given as a precaution against cancer recurrence, usually recommended to high-risk patients. Recent studies have shown that for stage II patients, adjuvant therapy does not increase overall survival, but there is a significant increase in progression-free survival after therapy [1]. Therefore, it is important to identify a subgroup of patients with the highest risk of relapse who can potentially benefit from adjuvant chemotherapy. The informatics team at Georgetown University has generated and analyzed multi-omics profiling datasets in stage II CRC patients with or without relapse to identify molecular signatures in CRC that may serve both as prognostic markers of recurrence, and also allow for identification of the subgroup of patients who might benefit from adjuvant chemotherapy.

## Methods and Results

Mass spectrum data from serum and urine samples of 40 CRC patients (20 relapse and 20 relapse-free samples) were collected and pre-processed using a standard pre-processing pipeline. Statistical group comparison was performed using t-test (Bio-conductor). A total of 77 differential metabolites in serum and 47 in urine were identified. Nearly 60% of differential metabolites in both urine and serum were identified using the in-house developed Metabolomics - Data Analysis and Annotation Pipeline (M-DAAP). Enrichment analysis revealed top enriched pathways, including 'Nicotinate and Nicotinamide Metabolism' (*P* = 0.007) in urine and 'Methionine Metabolism' (*P* = 0.002) in serum. A comprehensive catalogue of genomic alterations in the coding regions of 40 tumor samples was also generated by whole-exome sequencing to decipher the mutational landscape associated with CRC relapse. Enrichment analysis of the statistically significant genes from a gene-based association test revealed several pro-inflammatory and chromatin remodeling pathways, including 'BradykininR → STAT3 signaling' (*P* = 0.005); 'INO80 Chromatin Remodeling' (*P* = 0.013); and 'TNFR → AP-1/ ATF/TP53 signaling' (*P* = 0.03).

## Conclusions

Preliminary exome data analysis revealed proinflammatory signaling as the most significant pathway. Bradykinin (BK) is a potent pro-inflammatory mediator and, together with TNF-α (another pro-inflammatory cytokine implicated in CRC), has been observed in colonic myofibroblasts, leading to enhanced signaling of several downstream kinases involved in oxidative stress, tumor proliferation and metastasis [2]. Urinary metabolites revealed Nicotinate and Nicotinamide Metabolism as the top enriched pathway. Genes in this pathway have been shown to be differentially expressed in colon cancer cells and are also closely related to oxidative stress and inflammatory response [3] and thus support its potential biological role in colon cancer etiology.
